# Temperature-Responsive Adsorption and Hydration Control of a Wide-Gradient Retarder in Oilwell Cementing (40 °C–120 °C)

**DOI:** 10.3390/ma19081555

**Published:** 2026-04-13

**Authors:** Chong Wang, Jinlong Peng, Chunyu Wang

**Affiliations:** 1 Sinopec Huadong Oilfield Service Corporation, Yangzhou 225000, China; 2 College of Materials Science & Engineering, Nanjing Tech University, Nanjing 266580, China

**Keywords:** oil well cement, retarder, surface adsorption, thermal stability, wide temperature range, hydration kinetics

## Abstract

The robustness of cement slurry performance under extreme vertical temperature gradients is critical for ensuring cementing operation safety in ultra-deep wells. This study systematically investigates the interfacial behavior and hydration control mechanisms of a temperature-sensitive composite retarder, TL-2. Adsorption analysis via Total Organic Carbon (TOC) reveals that TL-2 exhibits unique non-isothermal adsorption characteristics, where its adsorption capacity slightly increases with temperature (40 °C–90 °C). This behavior overcomes the conventional limitation of drastic adsorption decline at elevated temperatures and serves as the physicochemical foundation for its wide-temperature adaptability. Performance evaluations simulated wide-temperature gradient conditions: TL-2 provided stable thickening times at 120 °C, and samples developed adequate compressive strength after 3 days of curing at lower temperatures (40 °C and 60 °C) following an initial 120 °C thickening simulation. Microstructural characterization (XRD, MIP) further elucidates the strength evolution logic across the gradient: in the lower temperature zone (40 °C–60 °C), adequate strength is established within 3 days through precise induction period control; meanwhile, at 120 °C, matrix densification is enhanced by promoting the well-crystallized tobermorite formation. The results demonstrate that TL-2 achieves a refined “buffering” effect on the liquid-to-solid transition through dynamic interfacial regulation, exhibiting superior wide-temperature adaptability across extreme thermal gradients (40 °C–120 °C) and providing essential technical support for the operational safety of ultra-deep well cementing.

## 1. Introduction

The escalating demand for hydrocarbon resources has accelerated the exploration and development of ultra-deep reservoirs, where downhole environments present unprecedented engineering challenges. In these settings, primary cementing operations frequently encounter bottom-hole temperatures (BHT) exceeding 130 °C and pressures surpassing 50 MPa [1,2]. A defining characteristic of ultra-deep well construction is the steep vertical temperature gradient along the long cement column [3,4,5]. Consequently, a single cement slurry system must maintain stable rheology and controllable thickening time at the hot well bottom while ensuring rapid early strength development at the significantly cooler top of the cement column, which may be 80 °C–100 °C lower than the BHT. Failures in managing this “wide-gradient” thermal regime often result in either catastrophic flash-setting during placement or indefinite set-retardation at the casing shoe, both of which jeopardize the long-term zonal isolation and wellbore integrity.

Historically, chemical retarders such as lignosulfonates [6,7], hydroxycarboxylic acids [8,9], and synthetic polymers [10,11,12,13,14] have been employed to manage cement thickening windows. However, these conventional additives are fundamentally constrained by their temperature sensitivity. The adsorption efficiency of standard retarders generally diminishes as the temperature increases from 40 °C to 80 °C, a phenomenon that limits their adaptability to extreme thermal gradients [15]. Most organic retarders exhibit a sharp decline in efficiency at elevated temperatures due to thermal degradation or desorption from the cement grain surfaces. For instance, lignosulfonates often lose their retarding capacity above 110 °C, while synthetic retarders may demonstrate abnormal gelation or a “step-like” increase in consistency when subjected to high thermal gradients. This unpredictable behavior stems from the competitive kinetics between the thermally accelerated cement hydration and the temperature-dependent adsorption–desorption equilibrium of the retarder molecules.

Concurrent with the challenge of hydration control is the dilemma of ensuring mechanical integrity across the entire temperature profile. At bottom-hole temperatures (>110 °C), silica flour is typically incorporated to prevent strength retrogression by promoting the formation of stable phases like tobermorite [16,17,18,19]. However, the upper cement column (40–60 °C) faces a contrasting risk: high retarder dosages required for bottom-hole stability often induce excessive retardation at lower temperatures, preventing timely strength development. Therefore, the critical scientific challenge lies in achieving a unified system that simultaneously secures mineralogical stability at high temperatures and strictly controlled induction periods at low temperatures, thereby balancing the conflicting kinetic requirements of the wide-temperature gradient [20,21].

The efficacy of a retarder is dictated fundamentally by its adsorption capacity on the mineral surfaces (primarily C_3_S and C_3_A) [22]. For a retarder to be “temperature-adaptive,” it must maintain a stable interfacial barrier or dynamically adjust its surface coverage to counteract the thermal acceleration of hydration. Most existing studies focus on the performance of retarders at discrete temperatures rather than their adaptability across a continuous wide gradient. This work introduces TL-2, a compound retarder specifically engineered for wide-temperature applications. We systematically evaluate the adsorption isotherms of TL-2 alongside its impact on the mineralogical evolution and pore refinement of cement-silica blends. By integrating macroscopic consistency data with microstructural evidence across a 40 °C–120 °C range, we elucidate the mechanism by which surface-active retarders regulate the liquid-to-solid transitions in ultra-deep well environments.

## 2. Materials and Experimental Setup

### 2.1. Materials

Class G oil well cement (CEM) and silica flour were used as the primary binding materials. The chemical compositions of the cement and silica flour, determined by X-ray fluorescence (XRF), are summarized in Table 1. The actual mineralogical composition of the CEM was determined via XRD/Rietveld quantitative analysis, consisting of 60.5 wt% C_3_S, 19.2 wt% C_2_S, 2.4 wt% C_3_A, 14.8 wt% C_4_AF, and 3.0 wt% Gypsum. Compared to theoretical Bogue calculations, this direct phase quantification provides a more reliable basis for analyzing the hydration transitions and the retarding effect of TL-2. Silica flour with a high SiO_2_ content (98.18 wt%) and an average particle size optimized for hydrothermal stability (Ca/Si ratio targeted at 1.0) was incorporated at a dosage of 35 wt% by mass of cement.

The TL-2 retarder used in this study is a composite formulation of 1-Hydroxyethylidene-1,1-diphosphonic acid (HEDP, analytical grade) and Sodium hexametaphosphate (SHMP, analytical grade). The stock solution was prepared at a total concentration of 13.6 wt%, with a mass ratio of HEDP to SHMP maintained at 1.2:1. The components were dissolved in deionized water and stirred thoroughly for 1 h to achieve a stable, homogeneous phase. This synergistic system was designed to balance the potent sequestration of calcium ions with effective particle dispersion, ensuring its efficacy in specialized cementing environments.

### 2.2. Sample Preparation

Cement pastes were prepared with a constant water-to-solid (W/S) ratio of 0.44. In accordance with API Spec 10A, the liquid retarder mass was deducted from the total water content to maintain precision. As shown in Table 2, the silica flour dosage was fixed at 35 wt% to maintain a Ca/Si ratio of 1.0.

The testing temperature was set between 40 °C and 120 °C to simulate the representative geothermal gradients of Eastern and Northeastern China and to verify the stability of TL-2 under large temperature differences.

### 2.3. Adsorption Measurement

The adsorption of TL-2 on cement grains was quantified using the solution depletion method. Cement slurries with varying retarder dosages (1% to 5% by weight of cement) were conditioned in a pressurized atmospheric consistometer at a target temperature (40 °C–90 °C) for 20 min. The pore solution was extracted using a vacuum filtration apparatus (LICHEN Scientific, Shaoxing, China). The concentration of residual retarder in the filtrate was measured using a Total Organic Carbon (TOC) analyzer (Vario TOC SELECT, Langenselbold, Germany). The adsorption capacity ($Q_{e}$, mg/g) was calculated based on the difference between the initial and residual concentrations. The adsorption tests were conducted in triplicate, and the average values were reported along with their standard deviations to ensure data reproducibility.

### 2.4. Performance Testing

- **Thickening Time**: Tests were conducted at 120 °C at a pressure of 47 MPa using an HTHP consistometer (Bassrett, Shenyang, China), following API RP 10B-2 standards [23]. The heating rate was maintained at 5 °C/min.
- **Compressive Strength**: Strength development was evaluated under two conditions: (i) Constant HTHP curing at 120 °C/21 MPa for up to 28 days; (ii) A cooling-cycle simulation where samples were heated to 120 °C for 20 min and then cooled to 40 °C or 60 °C for a 3-day curing period to simulate the ”top-of-cement” environment. The compressive strength of each cement slurry formulation was determined using five replicates at each hydration age. The results are presented as the mean ± standard deviation, ensuring the statistical accuracy and reproducibility of the mechanical performance data.

### 2.5. Microstructural Characterization

- **X-ray Diffraction (XRD)**: Quantitative phase analysis was performed on samples after specific curing ages. At each hydration interval, the hydration was terminated by the solvent exchange method using isopropyl alcohol. The samples were subsequently ground to a particle size below 45 μm and stored in a vacuum desiccator to prevent carbonation prior to XRD analysis. Patterns were collected using a Rigaku diffractometer (Rigaku Corporation, Tokyo, Japan) (Cu-Kα, 40 kV/40 mA) over a 2θ range of 5–80°.
- **Mercury Intrusion Porosimetry (MIP)**: For MIP measurements, representative samples were cut into small cubes (approximately 3–5 mm) and immersed in isopropyl alcohol for 48 h. After solvent exchange, the samples were vacuum-dried at 40 °C to remove the internal moisture while preserving the delicate pore structure. Pore size distribution and total porosity were measured using a GT-60 porosimeter (QUANTACHROME, Boynton Beach, FL, USA) (pressure range 0.14–206.84 MPa) on crushed samples (∼3 mm) to assess the densification of the hydration products.

## 3. Results and Discussion

### 3.1. Adsorption Behavior of TL-2

**Figure 1:**
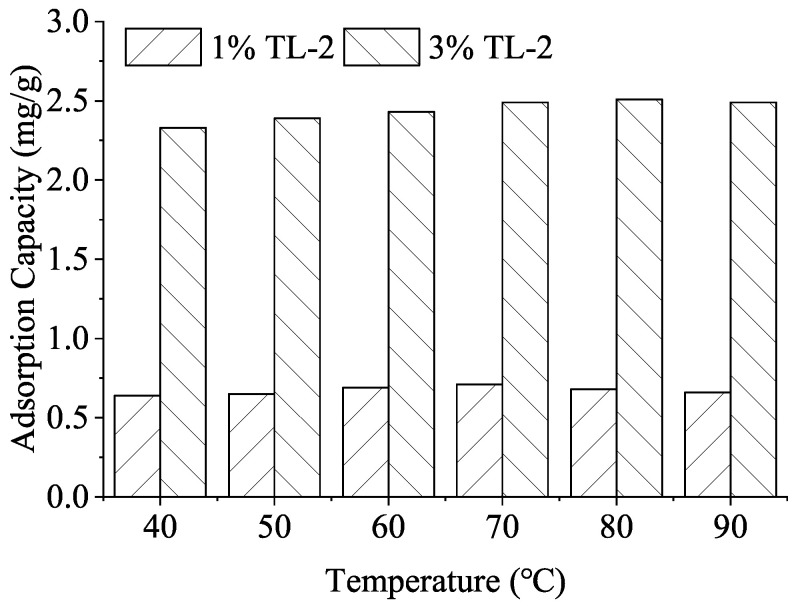
Influence of temperature on the adsorption of TL-2 on cement particles.

The adsorption of retarders on cement particles is the primary mechanism governing hydration kinetics. Figure 1 illustrates the adsorption capacity of TL-2 at temperatures ranging from 40 °C to 90 °C.

At a dosage of 1 wt%, the adsorption of TL-2 remains nearly constant at approximately 0.68 mg/g, indicating a stable coverage on the cement grain surfaces within this temperature range. More importantly, at a higher dosage of 3 wt%, the adsorption capacity exhibits a slight upward trend with increasing temperature, rising from 2.33 mg/g at 40 °C to 2.49 mg/g at 90 °C (Figure 1).

Typically, the adsorption of organic admixtures on mineral surfaces is an exothermic process, which causes the adsorption capacity to decrease as temperature rises. The stable or slightly increasing adsorption behavior of TL-2 is therefore a cornerstone of its temperature-adaptive performance. As higher temperatures accelerate the dissolution of cement phases and the nucleation of hydration products, the sustained or enhanced presence of TL-2 at the solid-liquid interface provides a continuous buffering effect. This prevents the rapid loss of retardation efficiency commonly observed in conventional systems, thereby ensuring operational safety across high thermal gradients.

### 3.2. Macroscopic Performance: Thickening Time and Compressive Strength

The operational reliability of cement slurry is primarily evaluated by its thickening time and its ability to develop mechanical strength after placement.

#### 3.2.1. Thickening Time at HTHP

**Figure 2:**
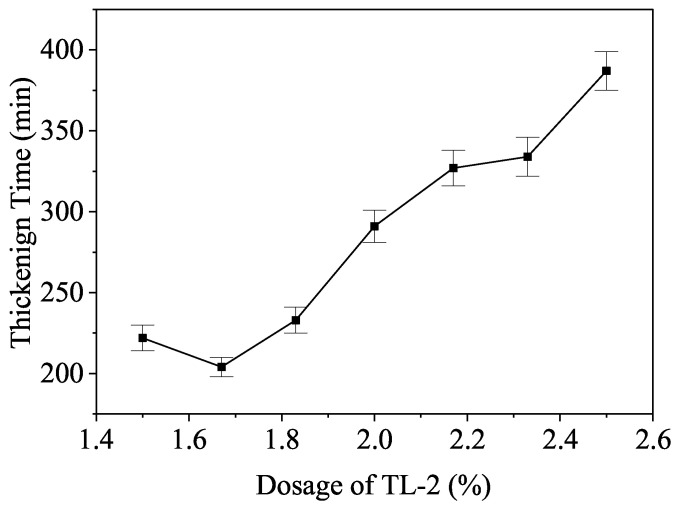
Influence of TL-2 dosage on thickening time at 120 °C.

As shown in Figure 2, the thickening time of the cement slurry exhibits a strong linear correlation with the retarder dosage at 120 °C. At 120 °C/47 MPa, an optimal retarder dosage of 2.0–2.5% provides a thickening time of approximately 387 min. These results confirm that TL-2 remains effective at high temperatures, providing sufficient safety windows for deep-well cementing operations.

#### 3.2.2. Compressive Strength Development

**Figure 3:**
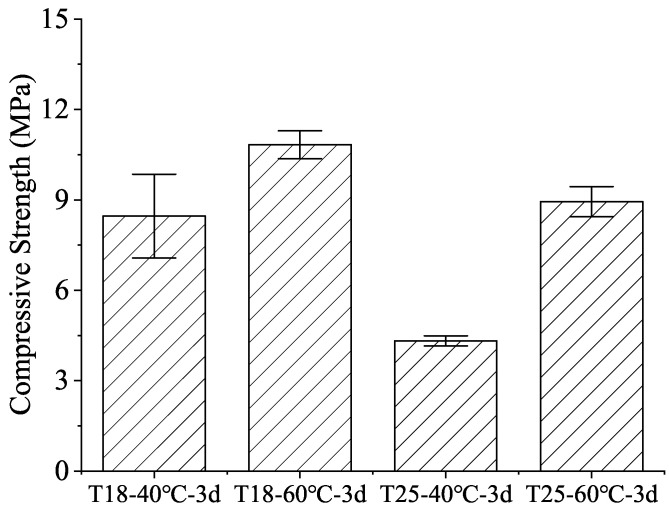
Compressive strength of cement pastes at 40 °C and 60 °C after a high-temperature cycle.

A critical challenge in wide-temperature cementing is the strength development at the “cool” upper sections of the cement column. Figure 3 displays the 3-day compressive strength of formulations T18 and T25 after a heating-cooling cycle (120 °C to 40 °C/60 °C). Despite the high retarder concentration, formulation T18 developed strengths exceeding 8 MPa at both 40 °C and 60 °C. Even the more heavily retarded T25 maintained strengths above 4 MPa. This indicates that while TL-2 is highly effective at bottom-hole temperatures, it does not lead to “over-retardation” or set-failure at lower temperatures, a common issue with traditional retarders.

**Figure 4:**
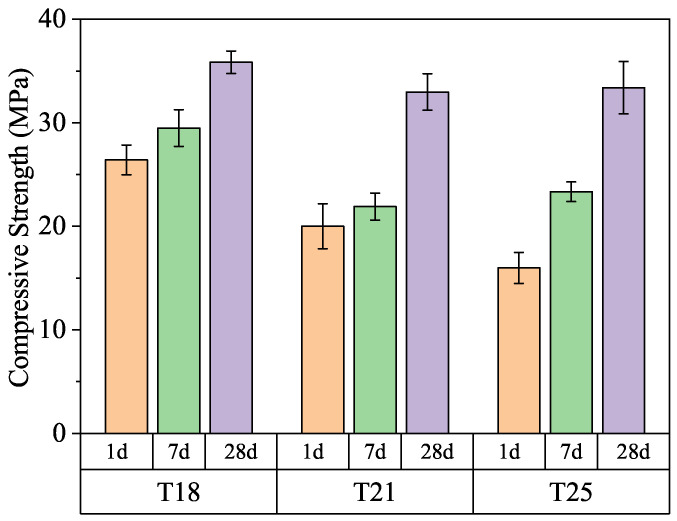
Long-term compressive strength development at 120 °C/21 MPa.

Furthermore, long-term curing at 120 °C (Figure 4) reveals that although the 1-day early strength decreases with increasing retarder dosage, the 28-day strengths for all formulations converge to approximately 33–36 MPa. This suggests that the presence of TL-2 influences the early hydration kinetics but does not impair the long-term structural integrity or the ultimate phase stability of the cement sheath.

### 3.3. Hydration Kinetics and Phase Evolution at Low Temperatures (40 °C–60 °C)

To understand the mechanical performance at the cooler top-of-cement sections, the phase evolution of T18 and T25 was monitored at 40 °C and 60 °C after the initial hot conditioning.

#### 3.3.1. Qualitative Phase Identification

**Figure 5:**
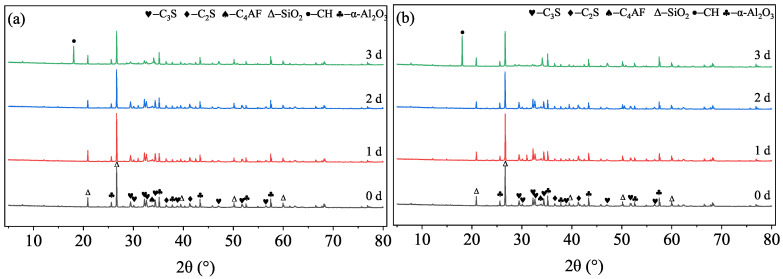
XRD patterns of T18 cured at 40 °C and 60 °C after a high-temperature cycle. (**a**) 40 °C; (**b**) 60 °C.

**Figure 6:**
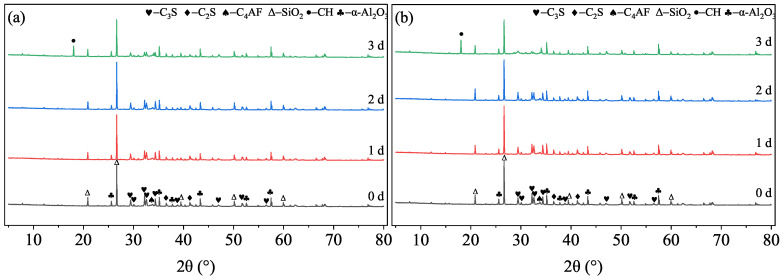
XRD patterns of T25 cured at 40 °C and 60 °C after a high-temperature cycle. (**a**) 40 °C; (**b**) 60 °C.

XRD patterns (Figure 5 and Figure 6) indicate that both formulations exhibit a significant hydration lag within the first 2 days. At 1 d and 2 d, the diffraction peaks are dominated by unreacted cement minerals (C_3_S, C_2_S, C_4_AF) and silica flour (SiO_2_), with negligible signals from hydration products. The characteristic peaks of portlandite (CH, Ca(OH)_2_) only emerge clearly after 3 days of curing. This delay confirms that the adsorption of TL-2 remains sufficiently potent at lower temperatures to suppress early hydration, which is essential to prevent premature setting during high-pressure placement.

#### 3.3.2. Quantitative Kinetics via Rietveld Analysis

Quantitative Rietveld analysis (Table 3 and Table 4) provides deeper insight into the hydration degree. For both T18 and T25, approximately 40%–45% of the original C_3_S remains unreacted at 2 d. However, between 2 d and 3 d, a sharp transition occurs: the C_3_S content in T18 drops from 40.32 wt% to 30.02 wt% at 40 °C, accompanied by a rapid increase in amorphous phase content (from 15.48 wt% to 23.91 wt%).

Similar trends are observed at 60 °C, where the acceleration is naturally higher, but the retarding effect of TL-2 remains evident, ensuring that the hydration products form a cohesive matrix only after the slurry has reached its target position. The eventual development of 4–8 MPa strength by 3 d (Figure 3) is thus directly attributed to this delayed yet robust accumulation of amorphous C-S-H and CH products once the retarder’s inhibitory threshold is surpassed.

### 3.4. Phase Stability and Microstructural Development at High Temperature (120 °C)

The performance of the cement sheath at the bottom-hole environment (120 °C) is dictated by the stability of hydrothermal products and the refinement of the pore network.

#### 3.4.1. Tobermorite Formation and Crystallinity

**Figure 7:**
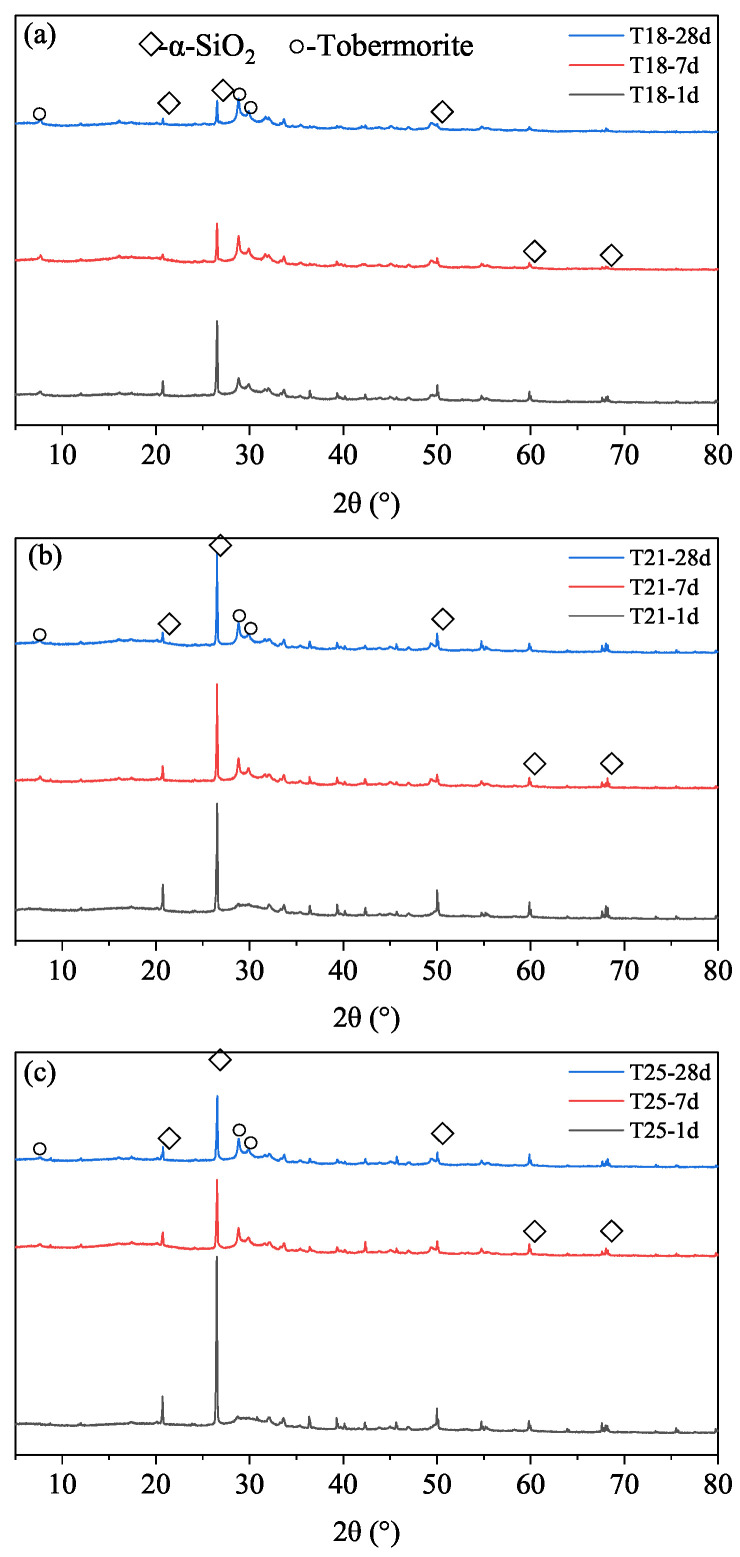
XRD patterns of HCPs with different TL-2 dosages cured at 120 °C and 21 MPa. (**a**) T18; (**b**) T21; (**c**) T25.

XRD patterns of HCPs cured at 120 °C (Figure 7) reveal that the hydration products are primarily composed of unreacted SiO_2_ and tobermorite. Notably, the phase composition remains stable from 1 d to 28 d, with no evidence of the brittle α-C_2_SH phase, confirming the effective role of silica flour in preventing strength retrogression at this temperature.

**Figure 8:**
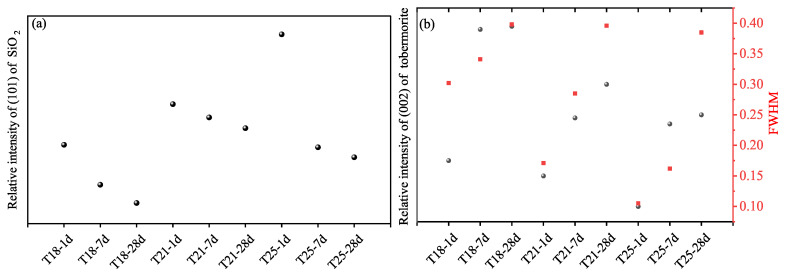
(**a**) Relative intensity and (**b**) FWHM of the tobermorite (002) peak as a function of time.

Analysis of the (002) reflection of tobermorite (Figure 8) shows that both the peak intensity and the full width at half maximum (FWHM) increase with curing age and with decreasing retarder dosage. According to Scherrer’s law, the increase in relative intensity and the narrowing of peaks (linked to micro-strain and crystal size) indicate that the crystallinity of tobermorite improves significantly over time [24]. Since no other crystalline products appear, the transformation of amorphous C-S-H into well-ordered tobermorite is the primary driver for the long-term strength gain observed in Figure 4. The retarder dosage influences the rate of this transformation at early ages but has a negligible effect on the ultimate mineralogical composition at 28 days [25,26,27].

#### 3.4.2. Pore Structure Densification (MIP)

**Figure 9:**
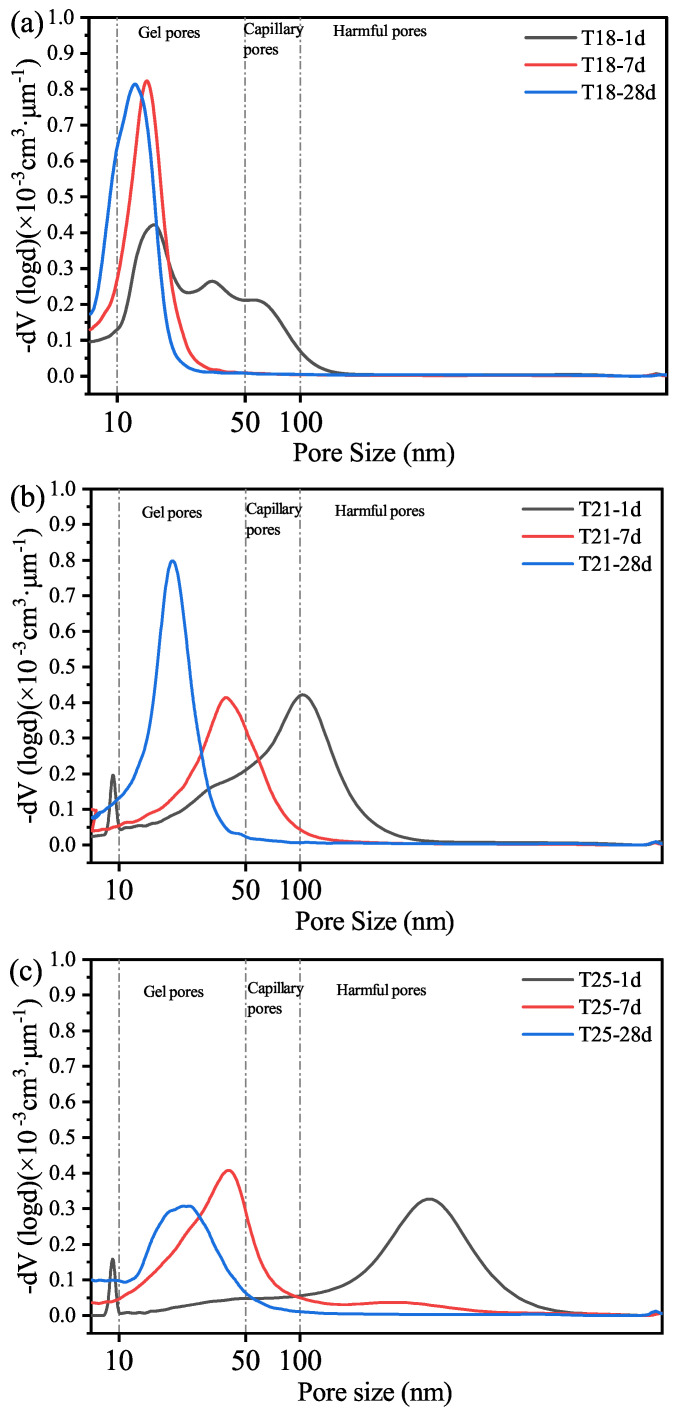
Pore size distributions (MIP) of HCPs with different retarder dosages at 120 °C. (**a**) T18; (**b**) T21; (**c**) T25.

The evolution of the pore system further supports the macroscopic results. As shown in Figure 9, increasing the curing age or decreasing the retarder dosage leads to a significant shift in the pore size distribution towards the gel pore range (<50 nm). At 1 d, the pore structure for highly retarded samples is relatively coarse. However, as hydration proceeds, the volume of gel pores increases, resulting in a denser matrix.

This densification is primarily due to the nucleation and growth of tobermorite and the corresponding reduction in unreacted silica volume. The transformation of a relatively porous HTHP hydration matrix into a dense structure dominated by low calcium-to-silica ratio crystalline products explains the high mechanical resistance and stability of the TL-2 retarded systems under deep-well conditions.

### 3.5. Thermal Regulation Mechanism of TL-2

The experimental data suggest a unique regulation model for TL-2. In typical organic retarders, surface adsorption is exothermic; thus, rising temperatures from 40 °C to 120 °C inevitably trigger molecule desorption, causing unstable slurry rheology and hydration “runaway” at the well bottom.

Conversely, TL-2 maintains a stable or slightly ascending adsorption capacity as thermal energy increases (Figure 1). This atypical behavior provides several advantages:

(a) Bottom-hole Stability (120 °C): The sustained surface coverage of TL-2 offsets the accelerated dissolution of polyaluminates and silicates. By preserving a robust interfacial barrier, TL-2 yields a predictable, linear dosage-thickening response (Figure 2), even under severe HTHP conditions.
(b) Top-of-Cement Set Control (40–60 °C): While the retarder remains active at lower temperatures, the quantitative XRD results clarify that this inhibition is a time-dependent induction rather than an irreversible blockage. Once the retarding molecules are integrated into initial hydration products (typically after 48–72 h), the C_3_S consumption accelerates rapidly, ensuring early strength development at the top of the cement column without the “non-set” risk inherent to over-retarded designs.

This temporal decoupling between early-age fluidity and late-age mineralogical stabilization allows TL-2 to manage the competitive kinetics of cement hydration across the diverse thermal zones of an ultra-deep wellbore.

## 4. Conclusions

This study evaluated the interfacial characteristics and hydration-retarding mechanisms of TL-2 under vertical thermal gradients (40 °C–120 °C). Key findings include:

(a) TL-2 adsorption on cement grains remains stable or increases with temperature (2.33 to 2.49 mg/g), representing a robust adsorption capacity that provides an inherent “buffer” against thermal hydration acceleration.
(b) Linear dosage control over thickening time was achieved at 120 °C, while top-section mechanical integrity was preserved, with strengths reaching 4–8 MPa within 3 days even after cooling from 120 °C.
(c) Quantitative Rietveld analysis linked the observed 48 h hydration lag at low temperatures to a controlled induction period, followed by rapid phase transition to amorphous C-S-H. At 120 °C, the slurry matured into a compact matrix of well-crystallized tobermorite with refined gel porosity, ensuring a final strength of 33–36 MPa without retrogression.

The temperature-responsive nature of TL-2 makes it highly suitable for long-column cementing, where slurry stability across large vertical temperature differences (40 °C–120 °C) is paramount. Future research will focus on the rheological properties and fluid loss control of TL-2 modified slurries under extreme thermal gradients to ensure a more comprehensive evaluation of their field applicability. Additionally, the long-term chemical durability and structural stability of the cement matrix in complex formation environments warrant further investigation to validate its integrity under specialized downhole conditions.

## Figures and Tables

**Table 1:** Chemical components of raw materials (wt%).

Oxide	SiO_2_	Al_2_O_3_	Fe_2_O_3_	TiO_2_	CaO	MgO	SO_3_	K_2_O	Na_2_O	LOI *
**CEM**	20.54	3.79	4.94	0.27	62.34	3.15	2.42	0.65	0.21	1.34
**Silica flour**	98.18	0.93	0.065	0.059	0.029	0.029	0.089	0.20	0.038	0.19

* LOI: loss on ignition at 1000 °C.

**Table 2:** Research Program for cement slurry characterization (wt%).

Mixture	CEM	Silica Flour	Retarder (TL-2)	Water
**T18**	65	35	1.95	42.05
**T21**	65	35	2.28	41.72
**T25**	65	35	2.71	41.29

**Table 3:** Mineralogical composition of T18 at 40 °C and 60 °C (wt%).

Curing Temp.	Age (d)	C_3_S	C_2_S	C_4_AF	CH	SiO_2_	Amorphous
–	0 d	44.03	14.16	11.73	0.87	25.01	4.20
**40 °C**	1 d	42.82	12.03	9.03	1.43	24.57	10.12
	2 d	40.32	8.18	8.87	3.13	24.02	15.48
	3 d	30.02	6.62	7.53	8.96	22.96	23.91
**60 °C**	1 d	41.32	12.63	7.38	2.25	23.07	13.35
	2 d	39.66	8.08	7.05	4.58	24.31	16.32
	3 d	26.35	7.66	6.80	9.15	23.68	26.36

**Table 4:** Mineralogical composition of T25 at 40 °C and 60 °C (wt%).

Curing Temp.	Age (d)	C_3_S	C_2_S	C_4_AF	CH	SiO_2_	Amorphous
–	0 d	45.14	15.81	11.43	0.12	25.30	2.20
**40 °C**	1 d	43.42	13.53	9.95	0.72	25.13	7.25
	2 d	40.92	10.97	9.77	0.32	24.47	13.55
	3 d	36.41	8.67	7.51	5.65	23.75	18.01
**60 °C**	1 d	43.60	12.11	9.75	0.20	24.96	9.38
	2 d	41.90	7.47	7.72	3.81	24.11	14.99
	3 d	33.88	6.05	6.98	7.90	23.43	21.76

## Data Availability

The original contributions presented in this study are included in the article. Further inquiries can be directed to the corresponding author.
